# Supplementary material for: Mitotic spindle positioning protein (MISP) preferentially binds to aged F-actin
Source: J Biol Chem. 2024 Apr 7;300(5):107279. doi: 10.1016/j.jbc.2024.107279 (PMC11101845; doi:10.1016/j.jbc.2024.107279)
Supplement: Supporting information [file mmc5.pdf]

SUPPLEMENTAL INFORMATION

Mitotic spindle positioning protein (MISP) preferentially binds to aged F-actin

E. Angelo Morales, Gillian N. Fitz, and Matthew J. Tyska

SUPPLEMENTAL FIGURES

Figure S1. MISP preferentially binds to aged actin filaments *in vivo* and *in vitro*. Related to Figure 1.

Figure S2. MISP preferentially binds to the ends of stabilized ADP-actin filaments. Related to Figure 2.

Figure S3. MISP assembles multi-filament parallel and antiparallel bundles. Related to Figure 4.

SUPPLEMENTAL VIDEOS

Video S1. MISP preferentially binds near the pointed ends of actin filaments. Related to Figure 2B. TIRF microscopy movie of freely diffusing EGFP-MISP (green) preferentially binding near the pointed ends of a tethered polymerizing actin filament (magenta). Scale bar = 3 μm.

Video S2. MISP preferentially captures actin filaments from the pointed ends. Related to Figure 3B. TIRF microscopy movie of immobilized EGFP-MISP (green) anchoring a freely diffusing actin filament near its pointed end. Scale bar = 3 μm.

Video S3. MISP forms two filament bundles in parallel and antiparallel fashion. Related to Figure 4. TIRF microscopy movie of MISP-driven bundling of two actin filaments (white). Left panel shows controls with no MISP. Middle and right panels show conditions with MISP, resulting in parallel and antiparallel bundling events, respectively. Scale bar = 4 μm.

Video S4. MISP bundles multiple actin filaments in parallel and antiparallel configurations. Related to Figure S3. TIRF microscopy movie of MISP-driven bundling of multiple actin filaments (black) landing on the field of view. Left panel shows control with no MISP. Middle and right panels show conditions with MISP, which result in parallel (‘P’) and antiparallel ('AP’) bundling events, respectively. Scale bar = 4 μm.

Figure S1

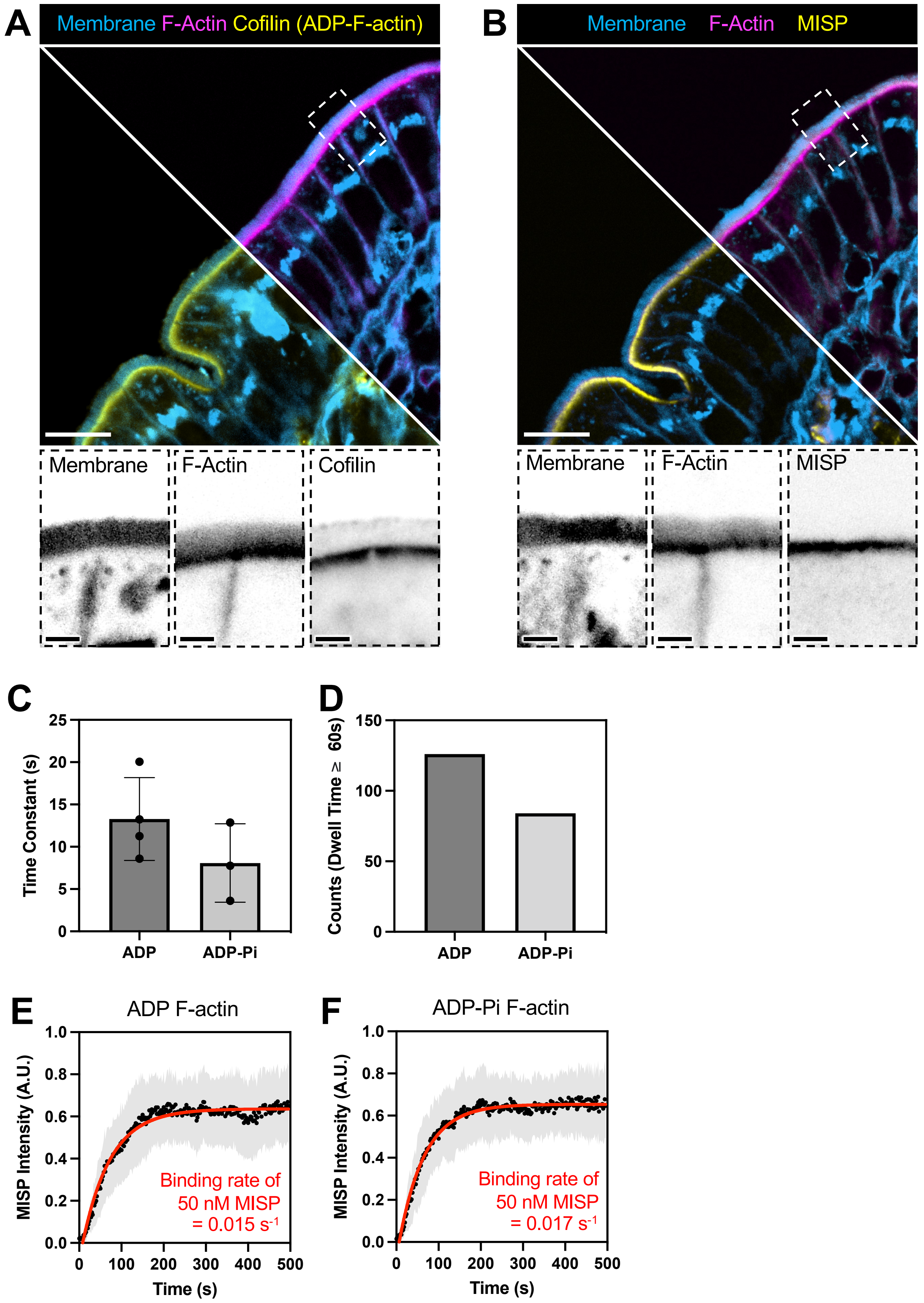
A
Membrane F-Actin Cofilin (ADP-F-actin)
Membrane
F-Actin
Cofilin
B
Membrane F-Actin MISP
Membrane
F-Actin
MISP
C
Time Constant (s)
25
20
15
10
5
0
ADP
ADP-Pi
D
Counts (Dwell Time ≥ 60s)
150
100
50
0
ADP
ADP-Pi
E
ADP F-actin
MISP Intensity (A.U.)
Binding rate of
50 nM MISP
= 0.015 s-1
Time (s)
F
ADP-Pi F-actin
MISP Intensity (A.U.)
Binding rate of
50 nM MISP
= 0.017 s-1
Time (s)

Figure S1. MISP preferentially binds to aged actin filaments *in vivo* and *in vitro*. Related to Figure 1. (A-B) Confocal images of separate frozen small intestinal sections stained for membrane with WGA (blue), F-actin with phalloidin (magenta), cofilin (yellow; panel A), and MISP (yellow; panel B). Each panel shows a split two-color merge (Scale bar = 10 μm). Bottom rows show inverted single channels for each marker in A and B (Scale bar = 2 μm). **(C)** Time constants of EGFP-MISP for each replicate used in Figure 1C-D. **(D)** Number of EGFP-MISP binding events lasting longer than 60 seconds in each nucleotide state of actin. **(E-F)** Accumulation of EGFP-MISP (50 nM) on immobilized F-actin (50 nM) enriched in ADP or ADP-Pi as a function of time. Exponential fits to fluorescence intensity data (red solid lines) were used to determine binding rates for 50 nM MISP under each condition. All data in each condition are representative of three independent experiments.

Figure S2

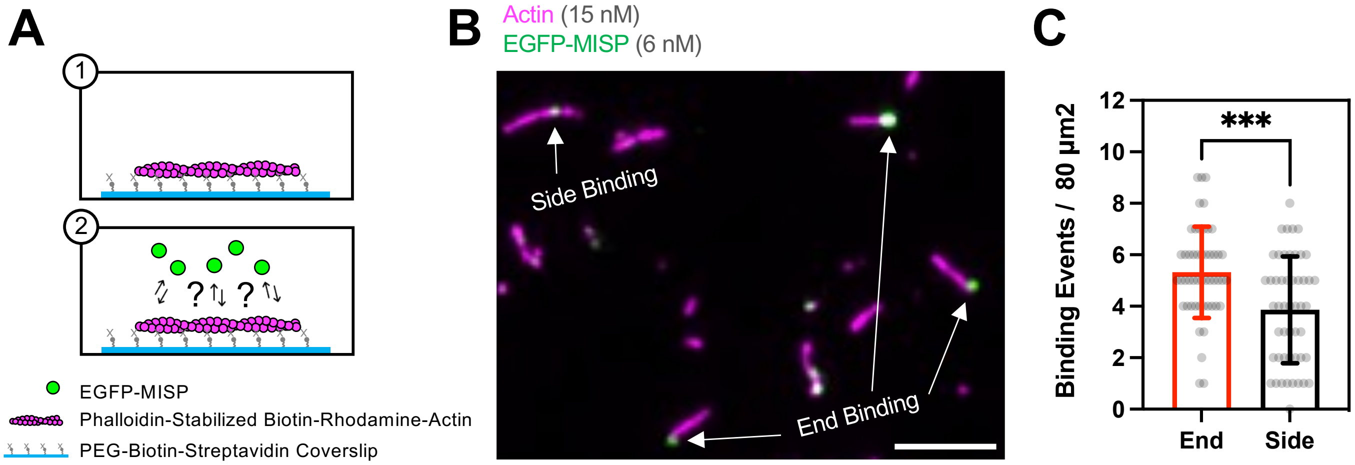

A
1
2
?
?
EGFP-MISP
Phalloidin-Stabilized Biotin-Rhodamine-Actin
PEG-Biotin-Streptavidin Coverslip
B
Actin (15 nM)
EGFP-MISP (6 nM)
Side Binding
End Binding
C
Binding Events / 80 µm2
12
10
8
6
4
2
0
***
End
Side

Figure S2. MISP preferentially binds to the ends of stabilized ADP-actin filaments. Related to Figure 2. (A) Cartoon schematic of the experimental setup (1-2). **(B)** TIRF microscopy image of phalloidin-stabilized biotin-rhodamine-actin filaments (magenta), and EGFP-MISP molecules (green). Scale bar = 5 μm. **(C)** Single binding events of MISP at the ends ("End") or at the side ("Side") of stabilized actin filaments from B. Each dot represents a single event. Bar plots and error bars denote mean ± SD. p value was calculated using the unpaired t test (***$p < 0.001$).

Figure S3

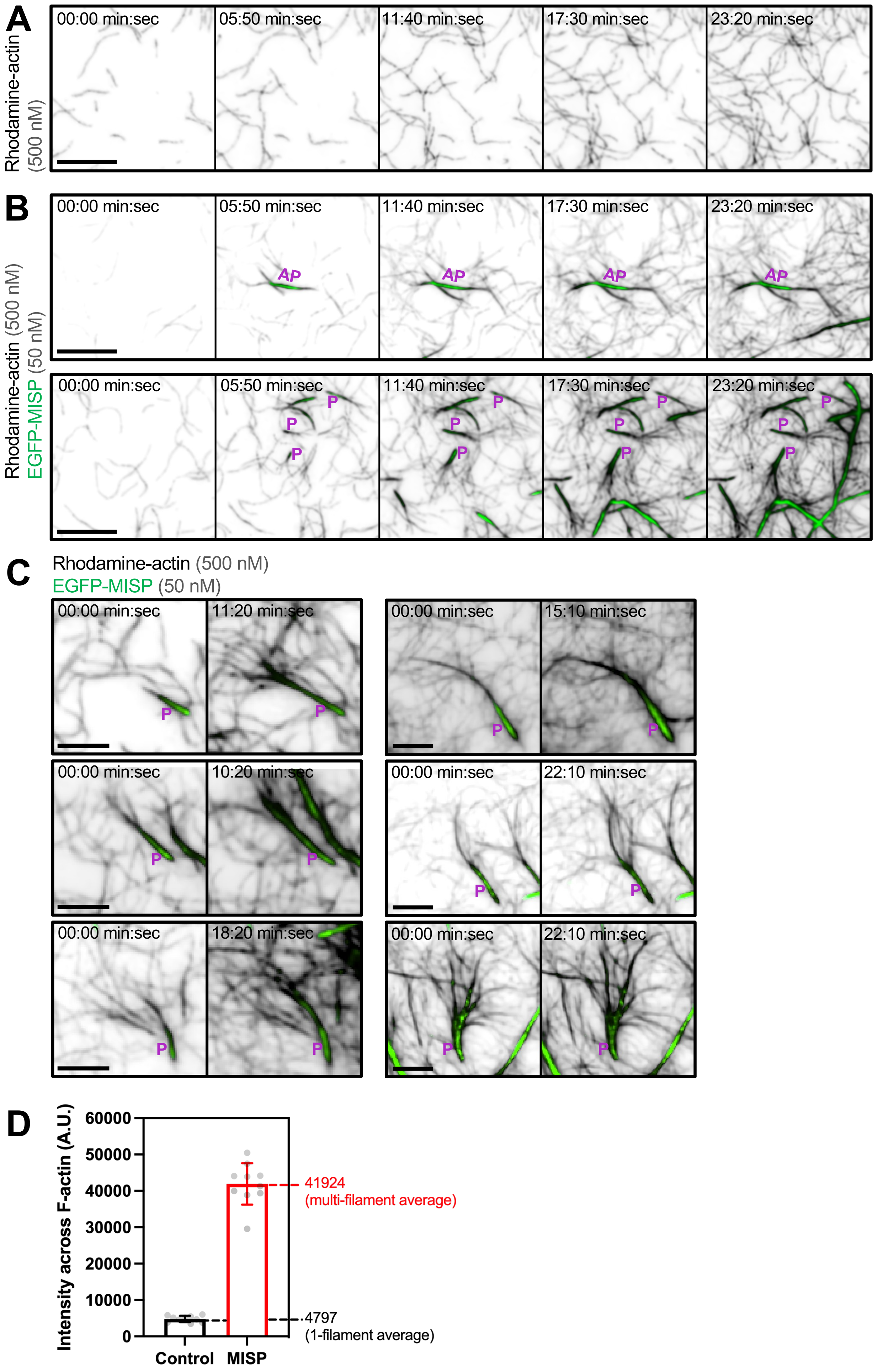
A
Rhodamine-actin (500 nM)
00:00 min:sec
05:50 min:sec
11:40 min:sec
17:30 min:sec
23:20 min:sec
B
Rhodamine-actin (500 nM)
EGFP-MISP (50 nM)
AP
P
C
Rhodamine-actin (500 nM)
EGFP-MISP (50 nM)
11:20 min:sec
15:10 min:sec
10:20 min:sec
22:10 min:sec
18:20 min:sec
D
Intensity across F-actin (A.U.)
60000
50000
40000
30000
20000
10000
0
Control
MISP
41924
(multi-filament average)
4797
(1-filament average)

Figure S3. MISP assembles multi-filament parallel and antiparallel bundles. Related to Figure 4. (A-C) TIRF microscopy montages of biotin-rhodamine F-actin (black), and EGFP-MISP (green). (A) Control experiment without EGFP-MISP. (B) Multi-filament antiparallel (middle row) and parallel bundling (bottom row) events driven by EGFP-MISP; AP = antiparallel bundling event; P = parallel bundling event. (C) Examples of multi-filament parallel bundling events before and after polymerization of their surface-bound actin filaments; P = parallel bundling event. Scale bar = 10 μm. (D) Mean intensity of single actin filaments (Control) and bundles of filaments (MISP) from movies shown in A-C. Bar plots and error bars denote mean ± SD.
